# A Randomized Comparison of Intravenous Versus Nebulized Routes for Administering Dexmedetomidine and Ketamine Combination to Facilitate Awake Fibreoptic Intubation

**DOI:** 10.7759/cureus.38322

**Published:** 2023-04-30

**Authors:** Chaitra Srinivas, Tanmay Tiwari, Ravi Prakash, Rati Prabha, Rajesh Raman, Zia Arshad

**Affiliations:** 1 Anesthesiology, King George's Medical University, Lucknow, IND

**Keywords:** nebulization, ketamine, dexmedetomidine, fibreoptic intubation, difficult airway

## Abstract

Introduction

Awake fibreoptic intubation is a technique used to secure the airway of patients who are having predicted difficult intubation. We compared two routes, intravenous and nebulized, for administering a combination of ketamine and dexmedetomidine to patients requiring sedation for fibreoptic intubation.

Methods and materials

After approval of the institutional ethics committee, 64 patients between 18 and 60 years belonging to the American Society of Anesthesiologists physical status I or II and having predicted difficult airway were randomized to receive study medications through either intravenous (group I, n = 32) or nebulized (group N, n = 32) routes. Group I patients were given a combination of ketamine and dexmedetomidine through intravenous route. Group N patients were nebulized with a combination of ketamine and dexmedetomidine. The time required for awake fiberoptic intubation was the primary outcome variable. In addition, sedation score, cough severity, patient tolerance, intubating condition, hemodynamic changes, recall of events and discomfort during intubation, and any adverse events in the perioperative period were also compared.

Results

Compared to group N, the time needed to intubate the patients was significantly less in group I (75.69 ± 10.83 versus 49.19 ± 3.60 seconds, p < 0.001). Observer assessment sedation/alertness score (p < 0.001), cough severity (p < 0.001), patient tolerance (p < 0.001), and intubating condition (p = 0.001) were statistically significant, all conditions being better in group I. Patient discomfort and recall of the procedure were statistically similar between the groups.

Conclusions

The efficacy of a combination of dexmedetomidine and ketamine through the intravenous route is better than the nebulized route for patients undergoing awake fibreoptic intubation.

## Introduction

Awake fiberoptic intubation (FOI) is the technique of choice for establishing airway access for patients with anticipated difficult intubation [1]. Drugs that suppress airway reflexes and maintain airway patency with minimal respiratory depression are preferred for this purpose. The drugs should also be cardio-stable and ensure patient cooperation, comfort, and amnesia. The most commonly used drugs are benzodiazepines, alpha-2 adrenergic agonists, propofol, and opioids which, when used in high doses, can produce respiratory depression [2].

Dexmedetomidine, an alpha-2 agonist, helps in sedation and analgesia without producing respiratory depression [1]. The reduced sympathetic tone produced by dexmedetomidine infusion is an additional advantage leading to decreased heart rate and blood pressure but produces xerostomia [3]. Ketamine has sympathomimetic action and produces excessive secretions, which can be useful to counteract the side effects of dexmedetomidine when used together. Ketamine, when used in low doses (4 µg/kg/min), reduces postoperative narcotic requirements and preserves the patient's ventilatory effort with analgesic properties [4]. In this trial, we compared the efficacy of intravenous and nebulized dexmedetomidine and ketamine combination for achieving better intubating conditions during FOI. The research question was whether nebulized dexmedetomidine and ketamine combination is superior to the intravenous combination of the same drugs for facilitating FOI in patients with anticipated difficult airways. It was hypothesized that nebulized dexmedetomidine and ketamine combination is superior to an intravenous combination of the same drugs for fiberoptic intubation of patients with anticipated difficult airways. The primary objective was to determine the difference in time taken to intubate the patients. The secondary objective was to determine the difference in intubating conditions, patient comfort, tolerance, cough severity, sedation level, amnesia, and additional propofol requirements.

## Materials and methods

This randomized, double-blind, comparative trial was started after obtaining clearance from the ethics committee of our hospital (reference No.: ECR/262/inct/UP/2013/RR-19). The study was done between December 2020 and August 2021. We included patients of either sex, aged 18-60 years belonging to the American Society of Anesthesiologists physical status I or II with predicted difficult airway (defined as mouth opening < three cm, thyromental distance < 6.5 cm, sternomental distance < 12.5 cm, or modified Mallampati class III/IV) scheduled to undergo elective surgery requiring endotracheal intubation. We excluded patients with coagulopathy, thrombocytopenia, epistaxis, a history of epistaxis, respiratory disease, cardiovascular disease, nasal pathology, and pregnant patients. All trial participants gave written and informed consent for participation in our trial. Using sequentially numbered, opaque, sealed envelopes, the recruited patients were randomly allocated to one of the following two groups. Microsoft Excel 365 (Microsoft, Redmond, Washington) was used for generating random numbers for randomization.

Patients in group I (n = 32) were given an intravenous infusion of ketamine (0.5 mg/kg), dexmedetomidine (1 µg/kg), and normal saline in a 20-milliliter syringe for 10 minutes. They were also nebulized with five milliliters of normal saline for 10 minutes.

Patients in group N (n = 32) were nebulized for 10 minutes with a five-milliliter mixture of dexmedetomidine (1 µg/kg) and ketamine (0.5 mg/kg) as well as normal saline. They were also given an intravenous infusion of 20 milliliters of normal saline for 10 minutes.

A preoperative visit was carried out one day before surgery. The patients were counseled for nasotracheal FOI. Patients were advised to fast six hours for solid food and two hours for liquids. Demographic data and airway details like inter-incisor gap, sternomental distance, upper-lip bite, neck movement, and thyromental distance were collected. The patients were unaware of the study group allocation. Study medications were prepared by an anesthesia technician in identical syringes. The technician was not involved in any other part of the trial. The observers were unaware of the study group allocation and study drugs administered to the patient. On the patient's arrival in the operation theater, peripheral intravenous access was secured, and baseline heart rate (HR), electrocardiogram, non-invasive blood pressure, and saturation (SpO_2_) were recorded. Xylometazoline (0.1%) was applied to both nostrils. Intravenous injection of 200 micrograms of injection glycopyrrolate and 4 milligrams of ondansetron were given, and oxygen was supplemented with a nasal prong at 4 liter/minute throughout the procedure. Two blocks were performed on both groups: superior laryngeal nerve block on both sides (with 2 milliliters of 2% lidocaine for each side) and transtracheal block (with 3 milliliters of 4% lidocaine). Study medications were administered according to the group allocated to the patient. Upon completion of drug administration through the specific route, the anesthesiologist assessed the sedation score using a modified observer assessment of alertness/sedation scale (OAAS) [5]. FOI was done by a consultant anesthesiologist having experience of more than five years and 100 FOIs. FOI was attempted only when the modified observer assessment of sedation was equal to or less than three [5,6]. If the OAAS was more than three, an injection of propofol was given in titrated doses to achieve an OAAS of three or fewer. An appropriately sized flexometallic endotracheal tube was placed over the flexible fiberoptic bronchoscope. The fiberoptic bronchoscope was inserted through one of the nostrils and directed into the patient's trachea. The endotracheal tube was guided into the trachea over the bronchoscope. The time taken to intubate the patient was the primary outcome variable. It was calculated as a time interval from the passage of the fiberscope tip through the nostril to the appearance of the capnography waveform.

The time needed for intubation and parameters like patient tolerance and cough severity, along with vital signs, was recorded by an assistant anesthesiologist. The intubating condition was graded by the anesthesiologist performing fibreoptic intubation. HR and systolic blood pressure (SBP) were assessed and noted at the following time points: baseline, every two minutes after the start of the drug for 10 minutes, fiberscope tip passage through nostril, nasopharynx, and glottis, and five minutes and 10 minutes after the intubation. After confirmation of endotracheal intubation by capnography, the patient was administered an intravenous injection of fentanyl (2 µg/kg), propofol (1.5 mg/kg), and vecuronium (0.1 mg/kg). Oxygen (50%)-air-isoflurane (0.5-1.5%) mixture and intermittent doses of intravenous vecuronium and fentanyl were used for the maintenance of anesthesia. After the end of the surgery, anesthetics were discontinued, and patients were extubated after the reversal of muscle relaxants. A visit was undertaken 24 hours after surgery to assess the recall of fiberoptic intubation and discomfort during the procedure [5]. OAAS [5], patient tolerance of the procedure [5,6], the severity of cough during the procedure [7], intubating conditions [8], patient discomfort [7], and recall of the procedure [6] are shown in Tables 1, 2.

**Table 1:** Grading of discomfort, cough severity, and intubating conditions during the procedure

Grade	Discomfort during the procedure	Cough severity	Intubating conditions
1	Nil discomfort	No cough	No hold-up or collision of endotracheal tube with glottis
2	Mild discomfort	Two or fewer coughs in sequence	Hold-up relieved by one rotation of the tube
3	Moderate discomfort or just tolerable	Three to five coughs in sequence	Hold-up requiring more than one rotation
4	Discomfort or completely intolerable	More than five coughs in sequence	Failed attempt at awake intubation

**Table 2:** Grading of sedation, patient tolerance, and amnesia of the procedure

Grade	Observer assessment sedation/alertness score (OAAS)	Patient tolerance	Recall of procedure
1	No response to mild prodding or shaking	No grimace	No recollection after the drug administration
2	Response to mild prodding or shaking	Minimal	Only partial recollection present
3	Response to name called out loudly or repeatedly	Mild	Full recollection of the procedure
4	Sluggish response to name called out in a normal tone	Moderate	-
5	Responds readily to name called out in a normal tone	Severe	-

Statistical analysis

Our trial had a power of 0.8 and an alpha error of 0.05. In the previous trial, the standard deviation for the time taken to intubate patients using FOI was 22.2 seconds [8]. To detect a difference of 30 seconds in the time taken to intubate the patient, 28 patients were needed in each study arm. To compensate for data loss and patient exclusions, 32 patients were recruited in each group. OpenEpi software (https://www.openepi.com/) was used to calculate the sample size. Statistical Product and Service Solutions software (IBM Corp., Armonk, NY) version 25.0 for Windows was used for all the statistical analysis. Continuous variables were analyzed using student's t-test and were summarized as mean ± SD. Discrete variables were analyzed using the Mann-Whitney U test and are summarized as median (interquartile range). Dichotomous variables were analyzed using Fisher's exact test and were summarized as numbers (percentages). A two-sided p-value less than 0.05 was taken to be statistically significant.

## Results

**Figure 1:**
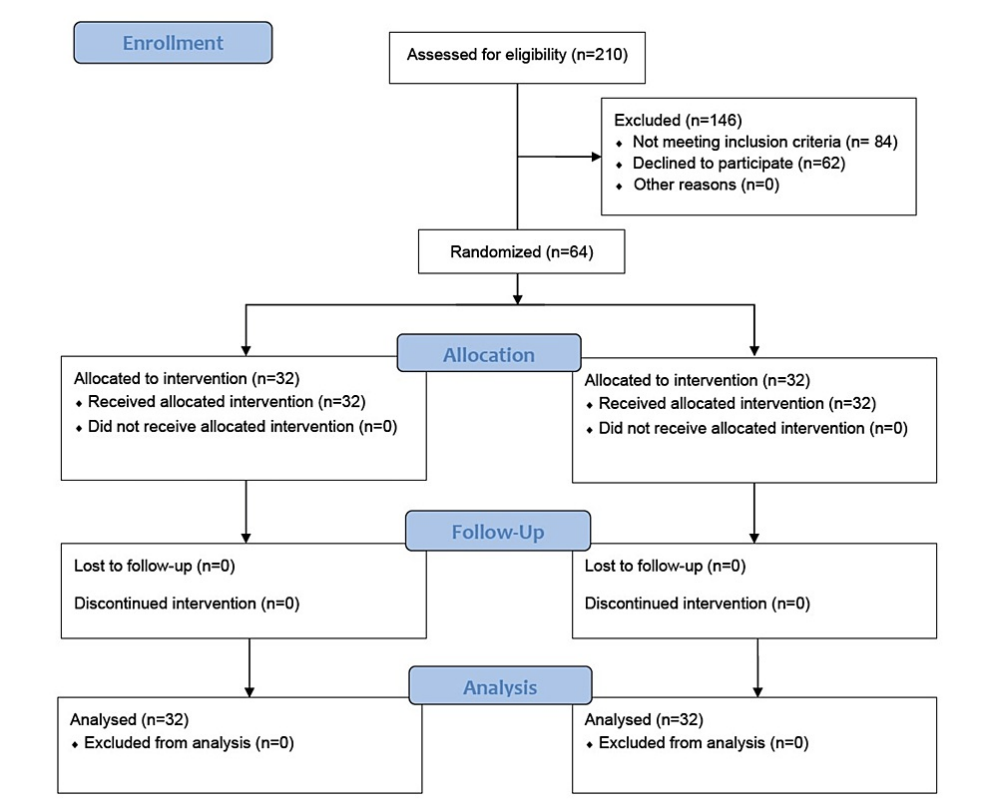
CONSORT diagram showing the flow of participants in the trial CONSORT: Consolidated Standards of Reporting Trials.

Consolidated Standards of Reporting Trials (CONSORT) diagram showing the flow of patients through the trial is shown in Figure 1.

Demographic and airway characteristics were comparable between both groups as shown in Tables 3, 4. Outcome measures are compared in Table 5. Intubation time, OAAS score, cough severity, patient tolerance, intubation condition, and propofol requirement were statistically lower in group I. Recall of the procedure and patient discomfort were statistically similar in both groups.

**Table 3:** Comparison of demographic and baseline characteristics of the two groups Data are presented as mean ± standard deviation and number (percentages). kg: Kilogram; m: Meter; BMI: Body mass index; ASA: American Society of Anesthesiologists physical status.

Characteristics	Group I (n = 32)	Group N (n = 32)	p-value
Age (years)	42.88 ± 9.98	47.16 ± 8.54	0.07
Gender (male/female)	26 (81.25%)/6 (18.75%)	27 (84.38%)/5 (15.63%)	0.740
Height (centimeter)	161.38 ± 6.02	163.72 ± 6.26	0.132
Weight (kg)	64.22 ± 4.56	62.75 ± 7.73	0.358
BMI (kg/m^2^)	24.72 ± 2.16	23.52 ± 2.85	0.062
ASA I/II	2 (6.25%)/30 (93.75%)	4 (12.50%)/28 (87.50)	0.391

**Table 4:** Comparison of airway characteristics of the two groups Data are presented as mean ± standard deviation and number (percentages). MPG: Modified Mallampati class.

Characteristics	Group I (n = 32)	Group N (n = 32)	p-value
MPG III/IV	6 (18.75%)/26 (81.25%)	8 (25.00)/24 (75.00)	0.545
Neck movement (adequate/inadequate)	29 (90.63%)/3 (9.34%)	32 (100.00%)/0 (0.00%)	0.076
Upper-lip bite test (positive/negative)	16 (50.00%)/16 (50.00%)	10 (31.25%)/22 (68.75%)	0.127
Mouth opening (cm)	1.60 ± 0.50	1.76 ± 0.64	0.279
Thyromental distance (cm)	5.72 ± 0.51	5.91 ± 0.32	0.082
Sternomental distance (cm)	10.84 ± 0.67	10.88 ± 0.91	0.876

**Table 5:** Comparison of the outcomes between the two groups Data are presented as mean ± standard deviation, median (interquartile range), and number (percentages). OAAS: Observer assessment sedation/alertness score. *Statistically significant.

Outcome	Group I (n = 32)	Group N (n = 32)	p-value
Time for intubation (seconds)	49.19 ± 3.60	75.69 ± 10.83	<0.001*
OAAS	2.00 (1.75-3.00)	3.00 (3.00-3.25)	<0.001*
Patient tolerance	1.00 (1.00-2.00)	2.00 (2.00-3.00)	<0.001*
Cough severity	1.00 (1.00-1.00)	2.00 (1.75-2.00)	<0.001*
Discomfort level	1.00 (1.00-2.00)	1.00 (1.00-1.00)	0.434
Intubating condition	2.00 (1.00-2.00)	2.00 (2.00-2.00)	0.001*
Recall of events (grade 1/2)	22 (68.75%)/10 (31.25%)	26 (81.25%)/6 (18.75%)	0.248
Requirement of propofol (yes/no)	32 (100.00%)/0 (0.00%)	0 (0.00%)/32 (100.00%)	<0.001*

**Figure 2:**
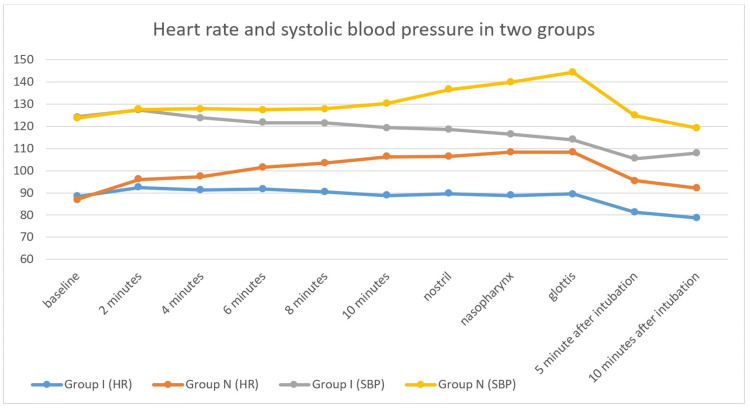
Comparison of heart rate (beats per minute) and blood pressure (mmHg) between the two groups SBP: Systolic blood pressure.

HR and SBP of the patients are depicted in Figure 2. The HR and SBP were statistically similar till 8 minutes of the study drug administration but were statistically higher in group I at all subsequent observation time points.

## Discussion

In this trial, we compared nebulized and intravenous routes for administering dexmedetomidine and ketamine for facilitating awake FOI in patients with an anticipated difficult airway.

It was observed that the intravenous route had a shorter time to intubation, lower OAAS score, less cough severity, required less propofol, and better patient tolerance and intubating conditions. There was no difference in the recall of events and patients' discomfort. The patients who were administered intravenous dexmedetomidine and ketamine had more stable hemodynamics than the nebulized route.

The guidelines for managing predicted difficult intubation clearly emphasize the use of awake FOI [9,10]. The route choice and the drug administered for facilitating awake FOI in patients with difficult intubation should balance patient cooperation and excessive sedation, which may compromise airway patency. Several drugs, including dexmedetomidine, midazolam, ketamine, and propofol have been used for this purpose. Intravenous is the most common route used for this purpose. However, the nebulized route is an attractive alternative as it can potentially avoid systemic side effects. Nebulized dexmedetomidine, lignocaine, and ketamine have also been used for other indications like pain relief, cough suppression, and pre-anesthetic sedative premedication for general anesthesia. This is the first study comparing intravenous and nebulized methods of administering dexmedetomidine and ketamine combination.

Preoperative nebulization with dexmedetomidine has been found to be better than placebo for suppressing sympathetic response to laryngoscopy and intubation [11]. Nebulized and intravenous dexmedetomidine were compared by Singh et al. for suppression of airway reflexes during direct laryngoscopy and tracheal intubation in 120 patients undergoing elective surgery [11]. Intravenous dexmedetomidine was found to suppress the response to laryngoscopy and intubation better than nebulized dexmedetomidine. Sancheti et al. compared the intravenous route of dexmedetomidine with a combination of nebulized and transtracheal injections of dexmedetomidine [12]. They found that dexmedetomidine delivered using a combination of nebulized and transtracheal routes had better patient satisfaction than the intravenous route. Dexmedetomidine combined with ketamine has been compared with intravenous dexmedetomidine alone for awake FOI in patients undergoing elective surgery [2]. The authors found that the use of both dexmedetomidine and ketamine was better than dexmedetomidine alone.

The sedative and analgesic effects of dexmedetomidine are mediated by stimulation of central 2 adrenergic receptors, primarily at locus coeruleus. The stimulation of central alpha-2 adrenergic receptors results in decreased systemic nor-epinephrine levels. This blunts the adrenergic sympathetic response to airway manipulation and prevents the rise in blood pressure and HR during the procedure [13]. Ketamine acts as an antagonist of the central N‑methyl‑D‑aspartate receptor and produces sedation and analgesia [14]. Ketamine causes sympathetic stimulation and increases blood pressure and HR. Their combination has the potential to provide stable hemodynamics due to opposing actions on the sympathetic nervous system.

The bioavailability of nebulized dexmedetomidine ranges from 65% to 82% and that of ketamine ranges from 20% to 40% [13,15]. This resulted in relatively lower plasma levels of dexmedetomidine and ketamine in patients of group N. We did not measure the plasma levels of the drugs in our trial. However, lower mean sedation level (OAAS score), a surrogate of the plasma level of the study medications, suggests that plasma levels were lower in patients nebulized with dexmedetomidine and ketamine.

The time to intubation was significantly shorter for patients who were administered study medications through the intravenous route than those through the nebulized route. This can be explained by the higher plasma levels achieved in group I, resulting in deeper levels of sedation. Furthermore, as ketamine and dexmedetomidine do not have a local anesthetic or analgesic action, nebulization did not have any effect other than the effect due to systemic absorption of the drugs through respiratory mucosa. Similarly, the cough severity, patient tolerance, intubating conditions, propofol requirement, and hemodynamic variables were better in group I than in group N. The trial findings of Sancheti et al. contradict the findings of our study [12]. This may be because the authors used an additional (transtracheal) route of dexmedetomidine administration in their study.

The current study's limitations include our trial's single-center design. Another limitation is that we did not measure the plasma levels of dexmedetomidine and ketamine. This would have allowed us to understand the implications of varying plasma levels on the clinical effect caused by the two drugs.

## Conclusions

We conclude that when compared to the nebulized route, the combination of dexmedetomidine and ketamine given through the intravenous route provides better conditions for awake fiberoptic bronchoscope-guided intubation in patients with anticipated difficult airway with faster intubation, better patient tolerance, and hemodynamic stability.
